# London Trauma and Cardiac Arrest Conference 2013

**DOI:** 10.1186/1757-7241-22-S1-A1

**Published:** 2014-07-07

**Authors:** F Bird, P Avery

**Affiliations:** 1 Barts Health NHS Trust, Bart's Health Hospital, London, UK; 2 London's Air Ambulance, The Royal London Hospital, Whitechapel, London, UK

## 

The seventh London Trauma Conference was once again held at The Royal Geographical Society in London from 10^th^–13^th^ December 2013. Over four days the latest innovations and best practice in cardiac, trauma and pre-hospital trauma care were presented and discussed. It opened with the London Cardiac Arrest Symposium, kindly sponsored by Zoll, with Dr David Zideman providing the Douglas Chamberlain Lecture; an insightful presentation into the world of Sports Medicine. Over the subsequent three days, delegates heard from internationally acclaimed speakers who addressed key questions relating to trauma, major incident management, and pre-hospital care. Keynote speakers included Professor Dan Davis, Prof Bryan McNally, Mr Michael Crumplin, Mr Justin Lewis and Professor John Pickard. The Norwegian Air ambulance hosted the Air ambulance and pre-hospital care day that reflected on the latest developments, debated best practice and speculated on the future of clinical work in this setting. On the final day, the Peter Baskett Lecture was given by Mr Michael Crumplin, who provided a fascinating over view of the developments in combat trauma care over the last six centuries.

Additional, breakaway sessions took place on each day of the conference. These included Resuscitative Thoracotomy, Practical Cardiac arrest and Neurotrauma masterclasses; Core topics in trauma, a Trauma Research Forum, and the annually anticipated evening of Stand up Science.

Prizes at the conference were awarded as follows;

The most inspiring clinical presentation was awarded to Dr Steen Barnung for his moving and impressive account of a multiple paediatric drowning incident that took place in Denmark.

Best posters: Dr Kate Crewdson and Dr Beth Healey

Stand up Science was won jointly by: Dr’s Maria Guerreiro and Hew Torrance.

The innovation prize was won by: Professor Hans Morten Lossius for his work on Pre-hospital stroke thrombolysis.

This year’s conference reminded delegates of the potential applications of new technology and innovation in pre-hospital, trauma and cardiac care but also the importance of ensuring that well established simple interventions are performed reliably and consistently. Once again the conference attracted speakers and delegates from 18 countries and continues to be a forum for learning network and debate. The eighth London conference will take place at the same venue between 9^th^–12^th^ December 2014.

This supplement provides a selection of seven extended abstracts written by speakers at the conference illustrating some of the diverse subject matter covered and also the scientific abstracts from oral and poster presentations made at the conference.

